# Dimeric Tubulin Modifies Mechanical Properties of Lipid Bilayer, as Probed Using Gramicidin A Channel

**DOI:** 10.3390/ijms25042204

**Published:** 2024-02-12

**Authors:** Tatiana K. Rostovtseva, Michael Weinrich, Daniel Jacobs, William M. Rosencrans, Sergey M. Bezrukov

**Affiliations:** 1 Program in Physical Biology, *Eunice Kennedy Shriver* National Institute of Child Health and Human Development, National Institutes of Health, Bethesda, MD 20892, USAbezrukos@mail.nih.gov (S.M.B.); 2 Center for Neutron Research, National Institute of Standards and Technology, Gaithersburg, MD 20899, USA; 3 Department of Biology and Biological Engineering, California Institute of Technology, Pasadena, CA 91125, USA

**Keywords:** amphitropic proteins, protein–lipid interactions, non-lamellar lipids, lipid packing stress, planar lipid membranes

## Abstract

Using the gramicidin A channel as a molecular probe, we show that tubulin binding to planar lipid membranes changes the channel kinetics—seen as an increase in the lifetime of the channel dimer—and thus points towards modification of the membrane’s mechanical properties. The effect is more pronounced in the presence of non-lamellar lipids in the lipid mixture used for membrane formation. To interpret these findings, we propose that tubulin binding redistributes the lateral pressure of lipid packing along the membrane depth, making it closer to the profile expected for lamellar lipids. This redistribution happens because tubulin perturbs the lipid headgroup spacing to reach the membrane’s hydrophobic core via its amphiphilic α-helical domain. Specifically, it increases the forces of repulsion between the lipid headgroups and reduces such forces in the hydrophobic region. We suggest that the effect is reciprocal, meaning that alterations in lipid bilayer mechanics caused by membrane remodeling during cell proliferation in disease and development may also modulate tubulin membrane binding, thus exerting regulatory functions. One of those functions includes the regulation of protein–protein interactions at the membrane surface, as exemplified by VDAC complexation with tubulin.

## 1. Introduction

Ion channels are known to be regulated by the mechanical properties of a lipid membrane [1,2,3,4,5,6]. In turn, these properties can be modulated by a variety of natural and synthetic amphiphilic molecules [7,8,9,10], many of which are recognized as pharmacologically active drugs. In this way, they affect ion channel behavior without direct interaction with the channel-forming protein. Much less is known about the effects of water-soluble or “amphitropic” proteins (APs), i.e., water-soluble proteins that reversibly associate with cellular membranes. APs are a subfamily of peripheral membrane proteins that interact directly with the lipid membrane rather than with intrinsic membrane proteins; therefore, this interaction is strongly influenced by membrane lipid composition [11,12].

Despite a significant amount of data demonstrating the binding of APs and water-soluble peptides to lipid membranes [13,14,15], the binding mechanism and its impact on lipid bilayer properties are still poorly understood. One of the obvious consequences of cytosolic AP binding to the cell membrane is that it changes the local concentration of the protein on the membrane and thus significantly alters the effectiveness of such protein interactions with membrane proteins and, in particular, ion channels. In other words, it is the local concentration of the protein that is physiologically important in its interaction with ion channels, not its bulk concentration in the cytosol.

Dimeric tubulin is one of the examples of a cytosolic AP which, in addition to its major role of being a building block of microtubules, also has been found to associate with different cell membranes and especially with mitochondrial membranes [16,17,18,19,20]. Dimeric tubulin is one of the most abundant cytosolic proteins. While total tubulin concentration in living cells is usually very high—up to 24 μM (in Xenopus oocytes and eggs [21])—the concentration of free dimeric tubulin is significantly lower (~8% of polymerized tubulin in HepG2 cells [22]) but could vary significantly throughout the cell cycle, depending on the type of cell [23]. Dimeric tubulin is a heterodimer with α-and β-subunits forming a water-soluble, compactly folded 110-kDa globule [24]. Both subunits have unstructured acidic C-terminal tails (CTT) exposed on the protein surface [25]. Extensive in vitro studies of tubulin binding to model liposome membranes in the 1980s revealed that this overall acidic protein could induce structural alterations of the lipid bilayer and that, surprisingly, tubulin–membrane interactions involve the hydrophobic membrane core [26,27,28]. The detailed structural mechanism of tubulin binding to the membrane was obtained three decades later using a combination of five independent biophysical techniques: neutron reflectometry (NR), surface plasmon resonance, electrochemical impedance spectroscopy, bilayer overtone analysis (BOA), and molecular dynamics (MD) simulations [29]. These measurements showed that tubulin binds peripherally, with the tubulin dimer axis oriented at approximately 60° relative to the membrane surface. Furthermore, all-atom MD simulations identified the amphipathic helix H10 of the α-tubulin subunit as the membrane-binding domain.

It was shown that the tubulin–membrane interaction is highly dependent on lipid composition [29,30]. Using confocal microscopy of giant unilamellar vesicles (GUVs) and fluorescently labeled dimeric tubulin, it was demonstrated that dimeric tubulin measurably adsorbs to the liposome membranes only in the presence of phosphatidylethanolamine (PE) in its mixture with phosphatidylcholine (PC) [30]. Later, using other biophysical techniques, the preferential tubulin binding to non-lamellar lipids with PE headgroups over those with PC headgroups was unambiguously confirmed [29]. MD simulations demonstrated the stable association of α-tubulin helix H10 with the DOPE membrane surface. The simplest model of tubulin–membrane interaction was suggested, where tubulin delivers its amphiphilic domain in the region of lipid polar headgroups. This model explains why tubulin binds more to the DOPE than to the DOPC membrane, considering that PE headgroups are smaller than PC [31] and thus provide more space between PE groups for tubulin anchoring.

PC and PE lipids are the main components of the mitochondrial outer membrane (MOM) [32], and it is noteworthy that even 40 years ago tubulin was shown to be bound to mitochondrial membranes by Bernier-Valentin et al. [19]. Later, it was found that tubulin binds to mitochondrial membranes in a wide range of cancerous and noncancerous human cell lines [16,33]. Carre et al. estimated the amount of mitochondria-associated tubulin as ~2% of total cellular tubulin. The physiological role(s) of tubulin binding to the mitochondrial membranes remained enigmatic [18] until a free dimeric tubulin was discovered to be a potent regulator of mitochondrial respiration [22,30,34,35,36].

The most ubiquitous protein in the MOM is the voltage-dependent anion channel (VDAC), which is known to be the main conduit for ATP, ADP, and other mitochondrial respiratory substrate transport across the MOM. The tubulin heterodimer was shown to effectively modulate VDAC conductance by partially and reversibly blocking VDAC reconstituted into planar lipid membranes (PLM) [34,35,37]. The tubulin-blocked state of VDAC is cation-selective and virtually impermeable for ATP [37], in contrast to the anion-selective and ATP-permeable VDAC open state. These results suggest that tubulin regulates the fluxes of metabolites and energy exchange between the mitochondria and other cellular compartments by modulating VDAC permeability. Experiments with isolated mitochondria [34] and human hepatoma cells (HepG2) [22,36] demonstrated that the VDAC–tubulin interaction is indeed functionally important for the regulation of mitochondrial respiration. It was also found that the on-rate of VDAC blockage by tubulin is 100 times higher when VDAC is reconstituted in PLM made from pure DOPE than in that made from DOPC [30].

These data led to the suggestion of a multistep model of tubulin–VDAC interaction, where the first step is saturable lipid-dependent tubulin binding to the membrane [30]. The second step is the voltage-dependent partial and reversible block of VDAC by a negatively charged tubulin CTT entering the net-positively charged channel pore. It was proposed that the effective concentration of tubulin on the surface of the MOM defines the on-rate of the final step of VDAC blockage.

Here, we study tubulin binding to planar lipid membranes using the gramicidin A (grA) channel as a sensor. This channel has proved to be a sensitive molecular probe of membrane mechanical properties [3,6,38,39,40]. The channel structure, a right-handed β^6,3^-helical dimer, is known to sub-angstrom resolution [41,42,43,44]. Formation of the grA conducting dimer of ~2.2 nm length [40] from two opposed grA monomers through the hydrophobic part of the lipid bilayer of ~4 nm thickness involves local bending of the two monolayers towards each other [45]. This creates a disjoining force on the channel structure, which is sensitive to the bilayer thickness, lipid packing stress, spontaneous curvature, lateral tension, elastic rigidity, and several other factors [2,3,38,46,47,48,49] and is one of the determinants of the conducting dimer lifetime (for a comprehensive review see [50]). Importantly, it was demonstrated that amphiphiles could affect the grA channel indirectly through the surrounding membrane by changing the lipid bilayer properties [5,46,49].

Here, we find that nanomolar concentrations of dimeric tubulin change the grA channel parameters and that this effect strongly depends on the lipid composition of the planar membranes. In membranes containing non-lamellar DOPE lipid, tubulin increases grA channel lifetime and, in addition, decreases channel conductance and induces characteristic fast conductance blockages. In pure lamellar DOPC bilayers, the effect of tubulin on grA was essentially negligible. The tubulin-induced increase of grA channel lifetime indicates that tubulin alters the membrane lipid-packing properties upon binding to DOPE-containing membranes, thus confirming a previously suggested model [29].

To further test the previously identified membrane-binding tubulin domain, we studied the effect of a synthetic peptide comprising the helix H10 of α-tubulin (residues A328–W346) on grA channel properties and demonstrated that this peptide does affect grA channel lifetime in DOPE membranes at 3–20 μM concentrations. Finally, using an independent method of BOA, we confirmed that the α-tubulin peptide measurably binds to DOPE-containing membranes but not to those made of pure DOPC.

Taken together, our data suggest that tubulin-membrane binding is not defined by the specific interaction of tubulin with lipid headgroups but exhibits rather unspecific binding depending on the degree of flexibility of the headgroups of the membrane surface. We also conclude that VDAC–tubulin interactions may be mediated by alterations in lipid bilayer mechanics in cells upon dynamic remodeling of mitochondrial membranes under mitochondrial fusion, fission, and many other cellular processes during cell proliferation in disease and development.

## 2. Results

### 2.1. Dimeric Tubulin Affects grA Channel Properties

**Figure 1:**
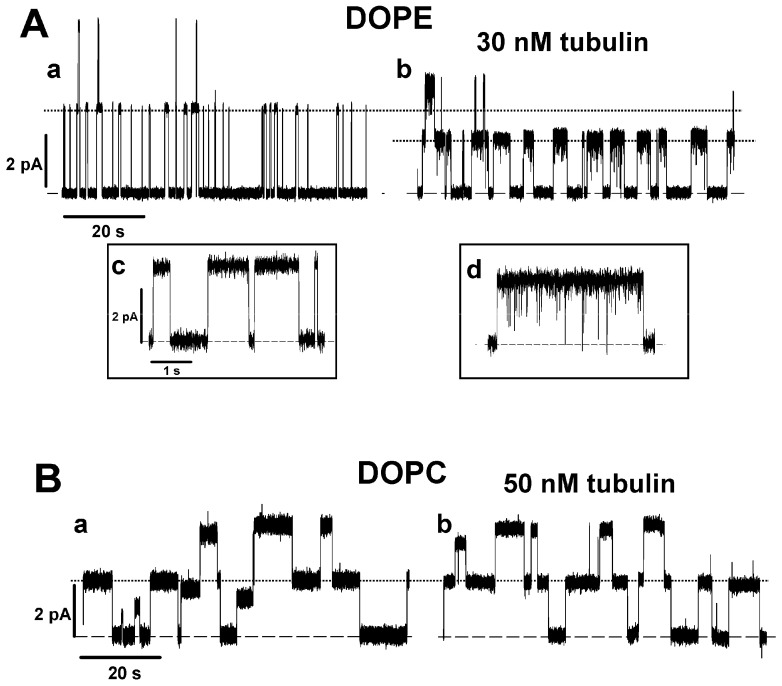
Tubulin increases the lifetime of grA channels and decreases their conductance in DOPE membranes but not in DOPC membranes: (**A**) Current traces of grA channels in DOPE membrane before (trace a) and after (trace b) addition of 30 nM tubulin. Tubulin notably increases grA lifetime and decreases channel conductance in the DOPE membrane. Tubulin also induces fast current flickering that can be better seen at a finer time scales in inset d in comparison with the control trace in inset c. (**B**) Current traces of grA channels in DOPC membrane before (trace a) and after (trace b) addition of 50 nM tubulin. The addition of 50 nM of tubulin does not appreciably change grA channel parameters in the DOPC membranes. The applied voltage was 100 mV. Tubulin was added to the *cis* compartment. Current records were digitally filtered using an averaging time of 10 ms. Dashed lines indicate zero current level and dotted lines indicate the currents through single grA channels. Here (and elsewhere) the medium consisted of 1 M KCl buffered with 5 mM HEPES at pH 7.4.

**Figure 2:**
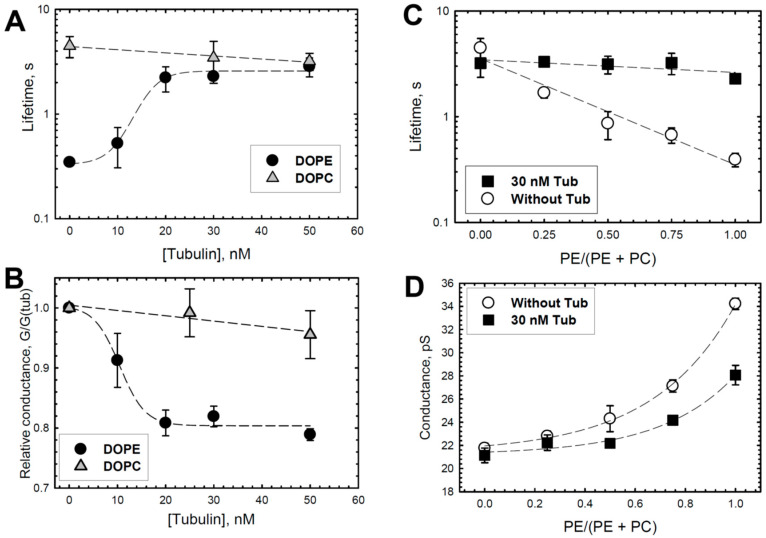
Effect of tubulin on the lifetime and conductance of grA channels, which depends on DOPE content in DOPE/DOPC mixture. In DOPE membranes, tubulin increases grA lifetime (**A**) and decreases conductance (**B**) in a dose-dependent manner that displays saturation at about 20 nM tubulin concentration. There is virtually no effect of tubulin on the channel lifetime and conductance in DOPC membranes. Channel conductance is given as its ratio in the presence of tubulin to that in the absence of tubulin. (**C**,**D**) Effect of 30 nM tubulin on the channel lifetime (**C**) and conductance (**D**) increases with PE content in the PE/PC mixture. Lines are drawn to guide the eye. Data are the mean values obtained in 3–5 experiments ± S.E. Experimental conditions are as in Figure 1.

To investigate the mechanism of the preferential binding of tubulin to DOPE versus DOPC membranes, we used the grA channel as a molecular probe. The representative current traces of grA channels in the PLMs made of DOPE and DOPC before and after the addition of tubulin are shown in Figure 1. It can be seen that in DOPE membranes, tubulin significantly increases the channel lifetime and reduces its conductance (compare trace a without tubulin and trace b with 30 nM tubulin in Figure 1A), whereas in DOPC bilayers these parameters are virtually unchanged in the presence of 50 nM tubulin (traces in Figure 1B). Figure 2 demonstrates that the effects of tubulin on the lifetime and conductance in DOPE membranes are dose-dependent and show clear saturation above 20 nM tubulin (Figure 2A,B). In contrast, in DOPC membranes, grA conductance and lifetime remain virtually unchanged (Figure 2A,B). Figure 2C,D show that the effect of tubulin on the channel was proportional to the DOPE content in PLM made of the DOPE/DOPC mixture. As we have shown previously, without tubulin, grA lifetime exponentially decreases with the increase in DOPE content in the DOPE/DOPC mixture ([39,51] and the open symbols of the control data without tubulin addition in Figure 2C of the present study). The addition of 30 nM tubulin increases the channel lifetime in pure DOPE membranes to almost the same value as in pure DOPC membranes (Figure 2A,C). Channel conductance changes less dramatically than the lifetime; however, the addition of 30 nM tubulin causes a consistent decrease in grA conductance that is proportional to the DOPE content in the DOPE/DOPC mixture (Figure 2D). Thus, tubulin interaction with PE-containing membranes could be monitored through the grA lifetime and conductance changes. In DOPC membranes, the changes are within the standard deviations of our measurements.

**Figure 3:**
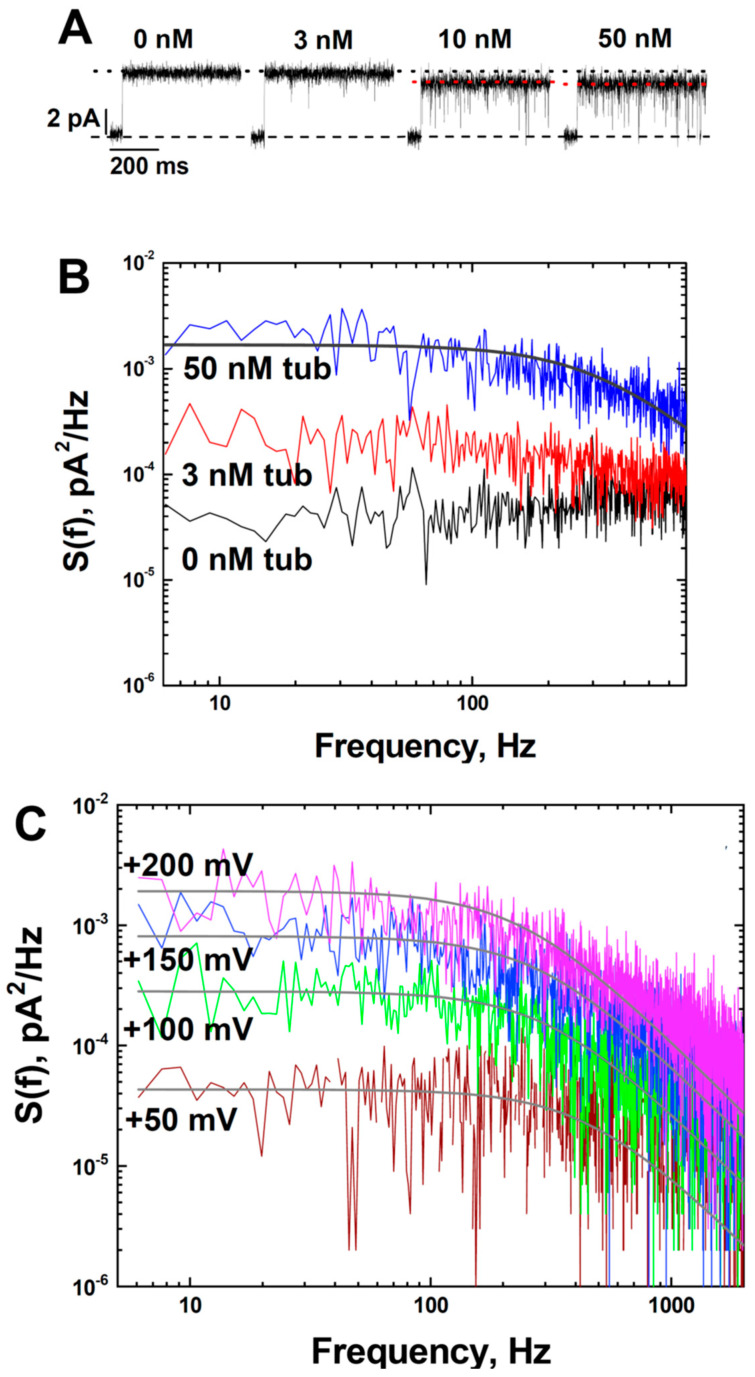
Tubulin induces fast flickering of the ionic current through grA channels in a dose- and voltage-dependent manner: (**A**) grA current traces were obtained at indicated tubulin concentrations in the DPhPC membrane at 200 mV applied voltage. The flickering increases with tubulin concentration and the corresponding decrease of the average conductance (black dotted line indicates conductance at 0 and 3 nM tubulin and red dotted line indicates conductance at 10 and 50 nM tubulin). Current records were filtered with a digital 8-pole Bessel filter at 1 kHz. (**B**) Power spectral densities of current fluctuations at 50 nM of tubulin (upper trace) can be approximated by a Lorentzian spectrum (smooth line through the data) with a corner frequency of *f_c_* = 600 Hz. (**C**) Tubulin-induced current fluctuations in the grA channel increase with applied voltage. Power spectral densities of current fluctuations of the single grA channel obtained at applied voltages as indicated in the presence of 50 nM tubulin. Solid lines are fitted with Lorentzian spectra. Current records were filtered with a digital 8-pole Bessel filter at 2 kHz. The medium consisted of 1 M KCl buffered with 5 mM HEPES at pH 7.4.

Another distinctive effect of tubulin on the grA channel was tubulin-induced current noise with a characteristic time in a range of hundreds of microseconds in DOPE-containing membranes. The fast-flickering current noise can be seen better in the *inset d* in Figure 1A, where the current traces before (*trace c*) and after (*trace d*) addition of 30 nM tubulin are shown at a finer time scale. To get an insight into the nature of tubulin-induced current noise, a quantitative analysis of current fluctuations was performed. The spectral density of the current noise generated by equilibrium fluctuations in conductance is known to scale as the applied voltage squared [52] and, therefore, application of a voltage as high as the planar membrane tolerates without breaking is desirable to achieve the best resolution. Taking advantage of the fact that PLM made from DPhPC can sustain a prolonged application of up to 200 mV potential and grA lifetime is significantly longer in DPhPC membranes compared to in DOPE membranes (~8 s vs. ~0.4 s, respectively, Table 1), we explored tubulin-induced noise using DPhPC membranes. It turned out that tubulin increased grA lifetime and induced fast flickering of the channel conductance, similar to its effect in pure DOPE membranes (Figure 3A and Table 1). These results are not surprising, considering that DPhPC is a unique lipid with properties that diverge from PC lipids, as measured by a series of independent methods [53].

Current traces in Figure 3A demonstrate that tubulin-induced fast flickering of the channel conductance increases with tubulin concentration, resulting in a corresponding decrease of the average channel conductance (dotted lines). Statistical analysis of tubulin-induced current fluctuations was performed by measuring their power spectral densities, which can be described reasonably well by Lorentzian spectra with a characteristic time of ~0.3 ms (Figure 3B). The weak dependence of this time on concentration (Supplemental Figure S4B) and the fact that the effect on integral conductance is relatively small suggest that this time represents the average time the channel spends in the tubulin-induced closed state [54]. The amplitude of the Lorentzian spectrum describing fluctuations at 200 mV was almost 100 times higher than that at 50 mV (Figure 3C), implying that the frequency of the tubulin-induced blockages increases with the increased applied voltage.

**Figure 4:**
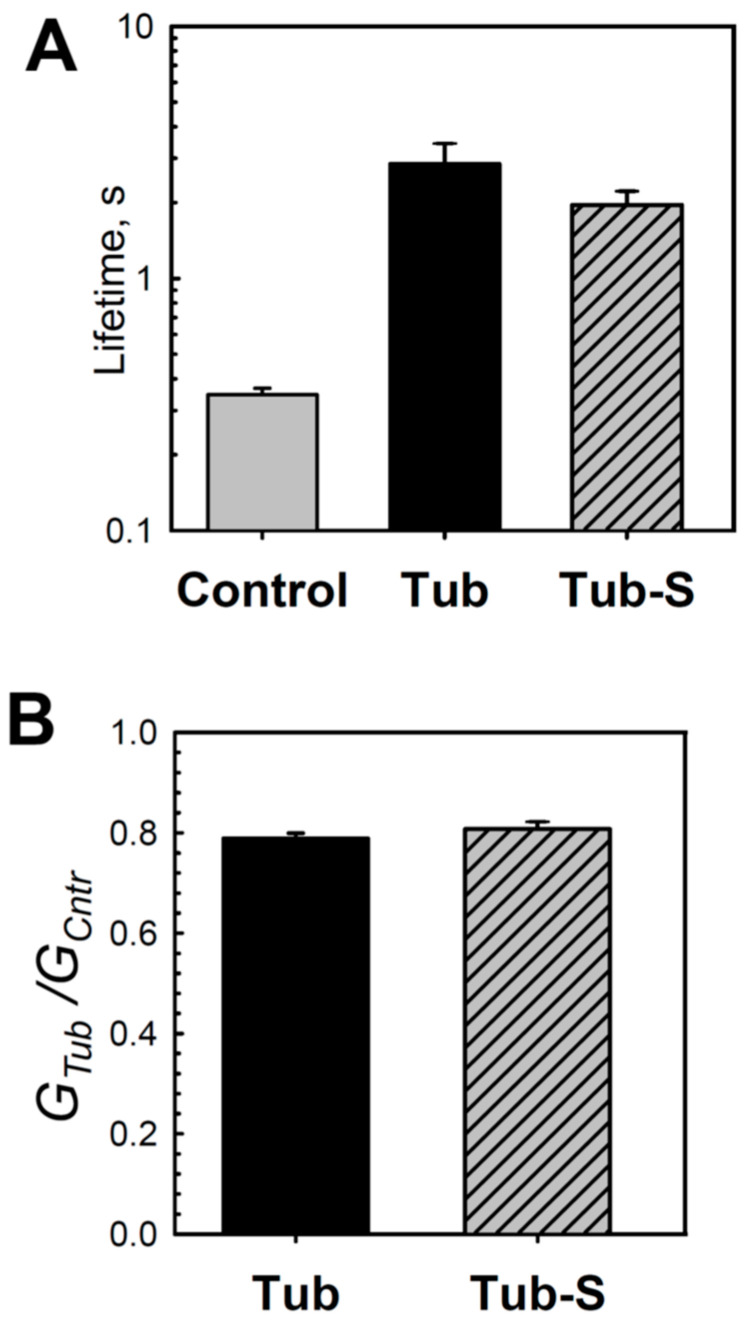
Tubulin-S changes the lifetime and conductance of grA channels in DOPE membranes. Similarly to tubulin, tubulin-S increases channel lifetime (**A**) and reduces its conductance (**B**) in comparison with the control. Channel lifetime and relative conductance (*G_Tub_*/*G_Cntr_*) were measured in the presence of 50 nM tubulin and 40 nM tubulin-S in the *cis* compartment. Data are the mean values obtained in 3–4 experiments ± S.E. Other experimental conditions were as in Figure 1.

Earlier, our group showed that CTTs of tubulin are required for its interaction with VDAC [34]. The conclusion was based on the observation that tubulin with proteolytically cleaved CTTs, tubulin-S, virtually did not block VDAC conductance. However, here we found that tubulin-S increased grA lifetime and decreased conductance in DOPE membranes in a manner that is similar to that for the intact tubulin (Figure 4). Interestingly, tubulin-S also induced a noticeable excess of current noise in grA channels (Supplemental Figure S1). These observations confirm our previous results that tubulin CTTs are not required for its interaction with the lipid membrane [29].

The fast blocking of grA conductance has been reported independently by a number of groups [55,56,57,58,59], with each group offering different explanations of this unusual behavior of the grA channel. Considering that the grA channel does not “gate” in the conventional sense [60], we attempted to further investigate the origin of this puzzling phenomenon. We decided to follow a model proposed by Armstrong and Cukierman [56] and Ring [58], where the intensity of grA channel flickering is related to the bilayer thickness. To test this hypothesis, we performed experiments with membranes formed from phosphatidylcholine with a C(22:1) unsaturated acyl chain, dierucoylphosphatidylcholine (diC(22:1)PC), whose hydrophobic thickness is 7.6 Å larger than that of DOPC, 44.3 and 36.7 Å, respectively [61] (Table 1). A hydrophobic mismatch between a thick PC bilayer and grA-conducting dimer should result in the formation of a distinctly large lipid-funnel-forming grA entrance that would lead to a less stable dimer and shorter channel lifetime. Indeed, in diC(22:1) membranes, channel lifetime decreased dramatically in comparison with DOPC membranes, from 4.5 s to 0.11 s, respectively (Table 1 and Supplemental Figure S2).

**Figure 5:**
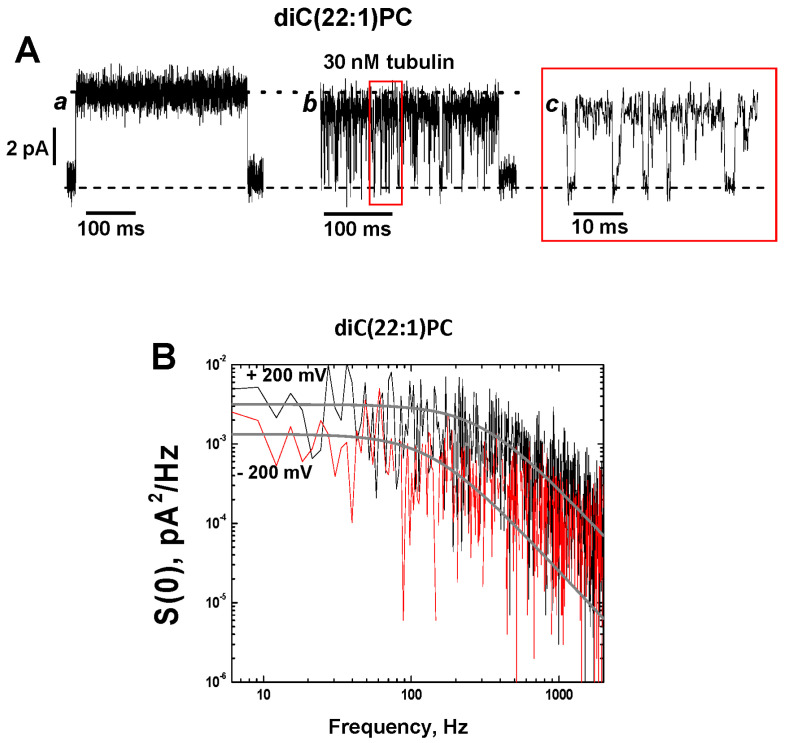
Tubulin-induced fast flickering of grA channels in diC(22:1)PC bilayers. (**A**) Current traces of a single grA channel in a diC(22:1)PC bilayer before (trace a) and after (trace b) addition of 30 nM tubulin to the *cis* compartment. The addition of tubulin induces rapid events of grA channel closure to a zero-current level (indicated by black dashed line), as shown in trace c at a finer time scale. The dotted black line indicates open channel conductance. The applied voltage was 200 mV. Current records were filtered with a digital 8-pole Bessel filter at 2 kHz. (**B**) Power spectral density of tubulin-induced current fluctuations, which depends on the polarity of the applied voltage. Solid gray lines represent the fits to Lorentzian spectra.

Similar to DOPC membranes, grA lifetime in diC(22:1) bilayers did not change in the presence of 30 nM tubulin (Table 1). However, tubulin still reduced grA single-channel conductance and induced current noise similar to what was observed in DOPE or DPhPC membranes (Figure 5). In diC(22:1) bilayers, grA conductance decreased by ~15% in the presence of 30 nM tubulin (Figure 5A and Table 1). Notably, the individual events of tubulin-induced blockage were well time-resolved in diC(22:1) membranes (Figure 5A, trace c), which allowed us to see individual events of conductance closure down to the zero-current level. These results suggest that the effect of tubulin on channel conductance and the stimulation of rapid conductance blockages might be of a different origin than the tubulin effect on grA lifetime. Most likely, the induction of rapid conductance closures which could be well time-resolved only in thick diC(22:1) membranes results in a decrease in average channel conductance in DPhPC- and DOPE-containing membranes. Accordingly, there is no decrease in grA conductance and no current fluctuations in the presence of tubulin with DOPC membranes.

**Table 1:** GrA channel lifetime and conductance in different PC and PE membranes without and with tubulin in 1 M KCl, pH 7.4.

			Without Tubulin	Without Tubulin	With 30 nM Tubulin	With 30 nM Tubulin
Lipid	Bilayer Thickness ^#^, nm	Area per Lipid, nm^2^	Lifetime, s	Conductance, pS	Lifetime, s	Conductance, pS
DOPC (C-8:1)	3.67 (*)	0.724	4.5 ± 1.0	21.8 ± 0.4	3.2 ± 0.9	21.1 ± 0.6
DOPE/DOPC (3:1) (C-18:1)	4.6 (**)	0.64	0.4 ± 0.05	34.2 ± 0.5	2.3 ± 0.1	28.1 ± 0.8
diC(22:1)PC	4.43 (*)	0.693	0.11 ± 0.01	19.1 ± 3.8	0.125 ± 0.08	16.4 ± 4.3
DPhPC C-18-(CH3)_4_	3.64 (***)	0.805	7.9 ± 0.4	22 ± 0.7	39.5 ± 3.3	18.9 ± 1.0

*—from Kucerka et al. [61]; **—from Rand et al. [62]; ***—from Tristram-Nagle et al. [53]; #—the phosphate-phosphate thickness.

The power spectral density of tubulin-induced current blockages demonstrates a pronounced dependence on the membrane lipid composition with the highest amplitude in the diC(22:1) membranes and asymmetry with respect to the sign of the applied voltage (Figure 5B and Supplemental Figure S3). In all experiments, the power spectral density was higher when positive potentials were applied from the side of tubulin addition (*cis* side in our experiments), regardless of the membrane lipid composition.

### 2.2. Probing Membrane Binding of Peptide-Mimicking α-Tubulin Binding Domain

**Figure 6:**
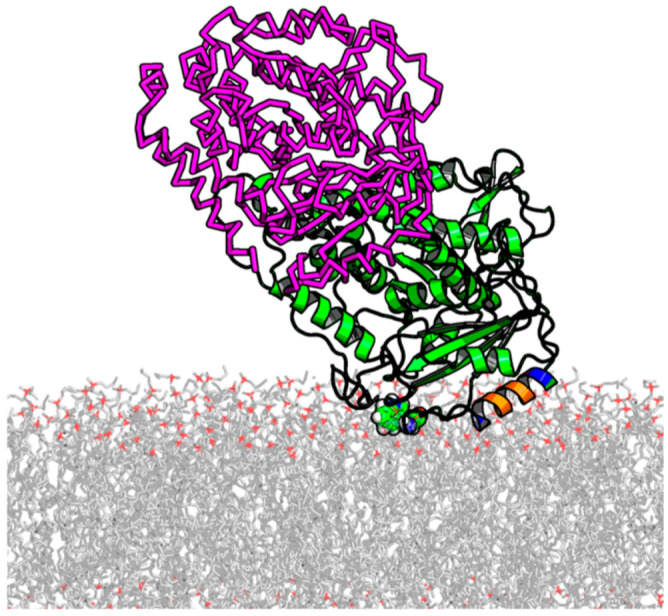
Illustration of stable α-tubulin binding to the DOPE membrane surfaces from ~800 ns of all-atom ANTON MD simulations. The insertion helical region (A330–W346) is colored according to chemical functionality (negative residues in red, positive residues in blue, and hydrophobic/aromatic in orange). The location of W346 is shown in sphere mode. β-tubulin, shown in the magenta-colored ribbon, is added based on RMSF alignment for α-tubulin in the dimer. MD simulations were performed for α-tubulin only. The unstructured C-terminal tails are not shown. Adapted with permission from Hoogerheide et al., *Proc. Natl. Acad. Sci. USA* (2017) [29]. Copyright (2017) National Academy of Sciences.

Previously, a membrane-binding tubulin domain was identified as the α-tubulin’s amphipathic helix H10 using a combination of five biophysical methods: SPR, electrochemical impedance spectroscopy, BOA, NR, and MD simulations [29]. It was shown that tubulin binding is strongly enhanced in membranes containing non-lamellar DOPE lipid [29,30]. Using NR in combination with long-lasting (over 880 ns) all-atom ANTON MD simulations, the orientation of the tubulin dimer at the membrane surface was determined, where the α-subunit binds preferentially to the membrane and the dimer tilt angle is ~60° [29] (Figure 6). Despite the set of different α-tubulin orientations on the surface, as found by NR and MD simulations, several binding residues located in the helix H10 (α-tubulin residues 325–337), including hydrophobic residues F343 and W346, were found to form long-lived contacts with lipids in MARTINI simulations. The hydrophobic amino acids in the binding region (indicated in bold) A_330_AIATIKTKRSIQFVDW_346_ were shown to interact with the hydrophobic core of the membrane, whereas the KTK_338_R_339_ fragment was stably coordinated by the phosphate moiety of the polar headgroups. It was concluded that the mechanism of tubulin–membrane association involves highly conserved hydrophobic residues on the helix H10 of α-tubulin. These results suggest that the binding helix of tubulin senses lipid-packing stress in the headgroup region and favors a more relaxed headgroup arrangement, as produced in membranes formed from nonlamellar lipids such as DOPE. Consequently, tubulin measurably affects the properties of the grA channel in DOPE-containing membranes, but not in those formed of pure DOPC.

**Figure 7:**
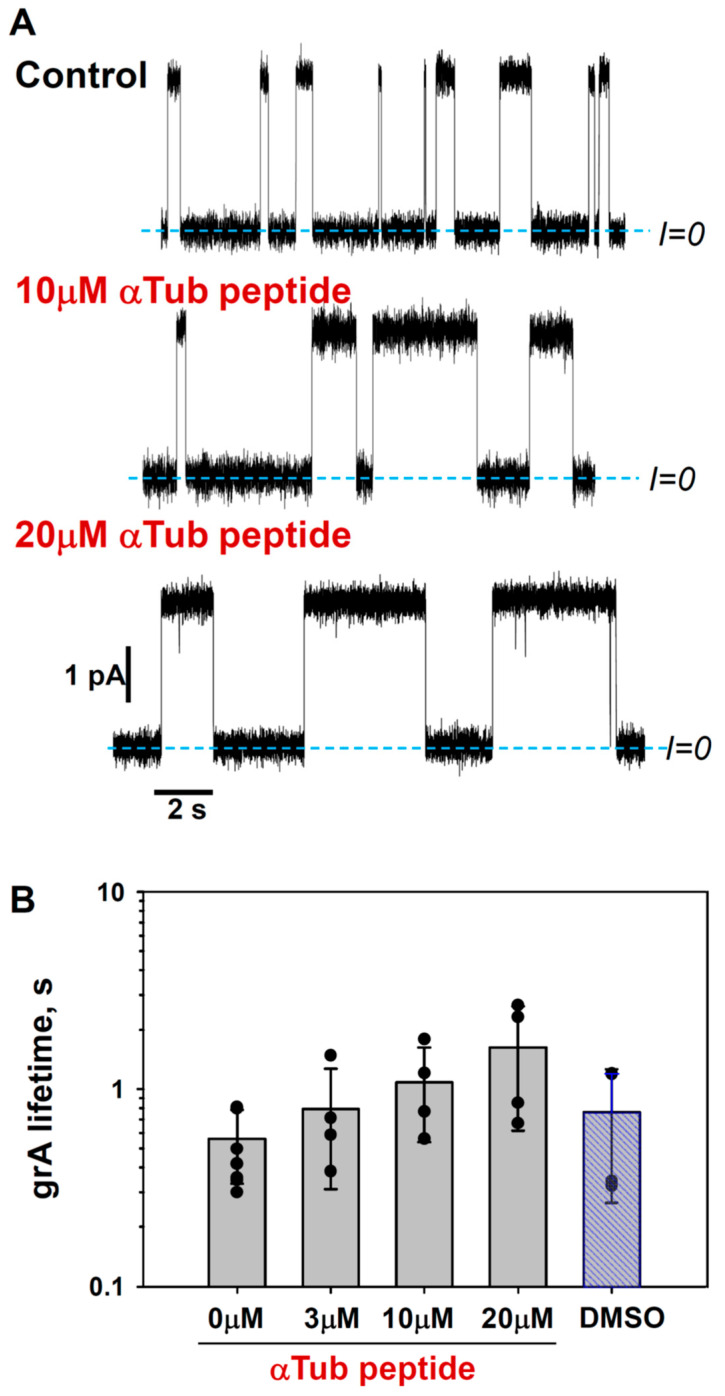
α-Tubulin peptide increases grA lifetime: (**A**) Current traces of grA channels in DOPE membrane before (Control) and after the addition of 10 and 20 μM of α-tubulin peptide to both sides of the DOPE membrane. Current records were filtered with a digital 8-pole Bessel filter at 1 kHz. Dashed lines indicate zero current level. The membrane bathing solution contained 1 M KCl buffered with 5 mM HEPES at pH 7.4. The applied voltage was 100 mV. α-tubulin peptide was dissolved in DMSO. (**B**) α-tubulin peptide increases grA lifetime in a dose-dependent manner. Bars and error bars are the mean and standard deviation from the mean; the symbols represent data points of 4 independent experiments. Control measurements with the addition of DMSO aliquots corresponding to 20 μM of α-tubulin peptide addition do not show the effect on grA lifetime.

To further test the membrane-binding propensity of the previously identified membrane-binding tubulin domain, we studied the effect of a synthetic peptide comprising the helix H10 of α-tubulin (residues A328–W346) on grA channel properties. The representative current traces of the channels in the presence of 10 and 20 μM of the α-tubulin peptide added to the *cis* side of the DOPE membrane are shown in Figure 7A. Like dimeric tubulin, the synthetic peptide also increases grA lifetime, but less significantly (~4 times increase) than tubulin (~10 times increase, Figure 2A) and requiring a much higher μM concentration range (Figure 7B and Supplemental Figure S5). In contrast to tubulin, the α-tubulin peptide does not affect channel conductance or induce conductance flickering in DOPE membranes. Changes in grA lifetime are indicative of the bilayer property changes caused by α-tubulin peptide binding to the DOPE membranes, thus supporting the results of MD simulations. The absence of the peptide effect on channel conductance suggests that the bulky tubulin dimer performs another type of interaction with the grA channel, which induces rapid conductance fluctuations.

**Figure 8:**
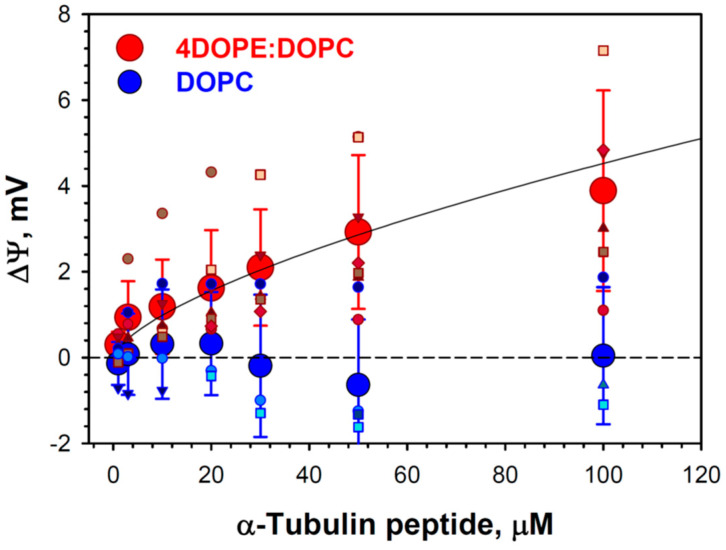
Binding curves for α-tubulin peptide interaction with DOPE and DOPC membranes, as measured by BOA. The transmembrane potential ΔΨ changes due to α-tubulin peptide binding to the DOPE/DOPC (4:1) (reddish symbols) membranes and does not change for DOPC (bluish symbols) membranes. Membranes were formed in 150 mM KCl buffered with 5 mM HEPES at pH 7.4. α-Tubulin peptide dissolved in DMSO was added to the *cis* side of the membrane. Aliquots of DMSO were correspondingly added to the *trans* side. Large red and blue circles and error bars are the mean and standard deviation from the mean for DOPE/DOPC and DOPC membranes, respectively; they represent data points of 7 individual experiments for DOPE/DOPC membranes and 6 experiments for DOPC membranes. The solid line is a fit to the binding equation with *K_d_* = 156 μM.

To compare α-tubulin peptide binding to DOPE with that to DOPC, we employed BOA measurements which allowed us to use the same system of PLM as in the grA experiments. BOA measures the second harmonic response of the lipid bilayer to the periodic excitation potential and thus permits measurements of the transmembrane potential ΔΨ induced by the electrical asymmetry between two lipid leaflets [63]. The addition of the α-tubulin peptide to the *cis* side of the membrane formed from the DOPE/DOPC (4:1) (mol/mol) mixture in 150 mM KCl produces a positive potential (Figure 8). There was no measurable change in ΔΨ up to 100 μM of peptide in the DOPC membranes. Because of the high variability between different experiments, we can only state that the binding constant is over 100 μM for DOPE membranes, which is more than 20 times higher than the *K_d_* obtained by the same BOA method for the whole tubulin dimer [29]. Nevertheless, these results confirm that α-tubulin peptide preferably binds to DOPE-containing membranes.

## 3. Discussion

**Figure 9:**
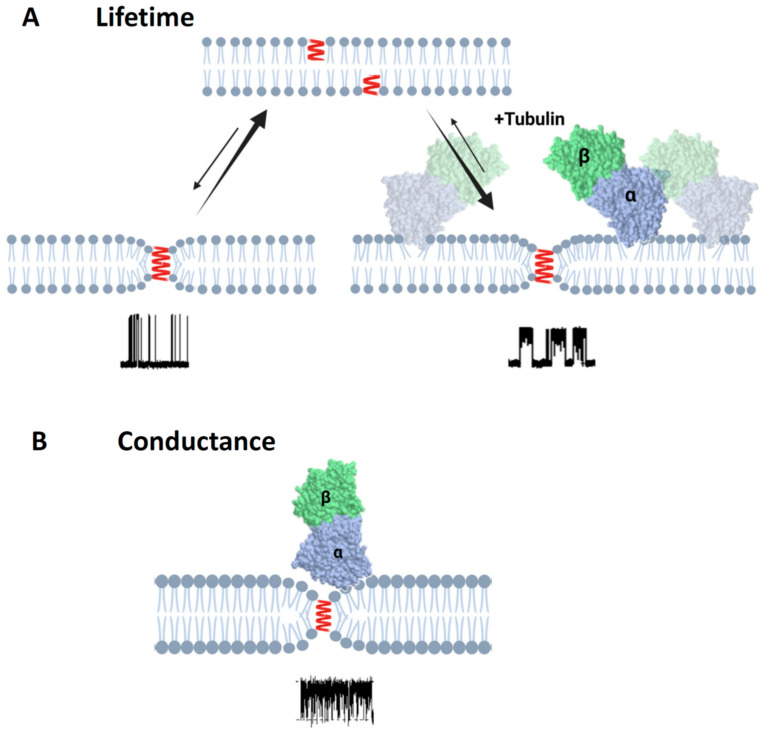
Schematics of the effect of tubulin dimer on grA lifetime and conductance: (**A**) Binding of α-β-tubulin heterodimers to the DOPE membrane reduces packing stress of the lipid tails, which is observed as the increase of grA channel (shown in red) lifetime. (**B**) In the case of diC(22:1)PC membranes, binding of the tubulin dimers is limited to the regions of membranes where headgroup packing is distorted by grA channel presence in the region of the lipid funnel forming the entrance to the channel. This limitation leads to the unchanged integral properties of the membrane and unchanged grA lifetime (Table 1); however, the localized binding is clearly manifested via transient channel blockages (Figure 5) from the bulky body of the tubulin dimer. Created with Biorender.com.

In our studies of the mechanism by which dimeric tubulin regulates the VDAC channel [30,34,35,37,64], we demonstrated that the lipid-dependent tubulin binding to the membrane is an essential step of tubulin interaction with VDAC reconstituted into planar lipid membranes. The MD simulations with the coarse-grained MARTINI force-field obtained with both α-tubulin and β-tubulin on pure DOPE and DOPC bilayers showed that α-tubulin resides on the surface of the DOPE membrane for the entire simulation time up to 1.2 μs [29]. By contrast, β-tubulin did not produce binding events longer than 50 ns. The most long-lived contact of α-tubulin with the membrane interface was found to involve helix H10, including the conserved residue W346. Here, we found that the net positive charge of the H10 helix corresponds to the increase of the positive ΔΨ measured in BOA experiments (Figure 8) with the α-tubulin synthetic peptide mimicking the H10 helix. These data support the previous idea that dimeric tubulin binding is governed by the α-helical amphiphilic domain of α-tubulin, which interacts preferentially with the membrane in the region of lipid polar headgroups (Figure 6) [29]. The above model explains why tubulin binds more readily to the DOPE than to the DOPC membrane. Due to the redistribution of the packing forces towards lipid hydrocarbon chains in the case of smaller PE headgroups [65], the latter are more flexible and are able to better adjust to the interacting tubulin domain (illustration in Figure 9A). This flexibility provides the conditions for stronger tubulin α-helix anchoring.

The efficiency of full-length tubulin in affecting grA lifetime differs from that of the α-tubulin peptide mimicking the H10 helix by three orders of magnitude. Such a difference in effective concentrations could be due to additional contributions to tubulin-membrane interaction from the rest of the tubulin’s body; this could explain both changes in the binding affinity and the effect of each bound item on the membrane properties. Another possibility is that of certain differences in the fine helical structure when it is a part of a tubulin molecule compared to the structure of a free peptide. It is unknown whether membrane-bound α-tubulin peptide maintains its helical structure or not, and the role of lipids in this process is also unknown. The ability of certain relatively small peptides to change their structure upon binding to lipid membranes is well described; the two best-studied examples of such peptides are the pH (low) insertion peptide (pHLIP) [66] and α-synuclein [67]. The intrinsically disordered α-synuclein polypeptide is disordered in solution but does have three distinct regions upon binding to membrane surfaces [68]. Notably, the adapted α-synuclein structure on the membrane surface depends on lipid composition: α-synuclein forms α-helical structure when interacting with anionic lipids, but remains unstructured on neutral PC membranes [69].

The effects of structurally and functionally diverse amphiphiles on bilayer properties have been described in the literature by the changes in a single parameter, a “phenomenological bilayer spring constant”, that could be directly derived from the changes in grA lifetime [46]. Here, using grA as a molecular probe of bilayer mechanics, we demonstrate that the amphitropic protein tubulin affects bilayer properties. Formally applying the terminology of Lundbaek et al. [46], we can conclude that tubulin decreases the phenomenological bilayer spring constant, which results in a substantial increase in grA lifetime. Tubulin reduces bilayer deformation energy contributions at grA dimer formation. A statistical model describing the general effects of membrane-deforming amphiphiles on membrane properties predicts the increase in grA-conducting dimers’ lifetimes as well as their number [49]. The authors concluded that if the membrane-binding amphiphile has a stronger affinity towards a particular lipid type (DOPE as in the case of tubulin), the amphiphile would amplify grA activity particularly in these lipid compositions. Both grA and BOA experiments with the synthetic peptide whose sequence corresponds to the previously identified helix H10 of α-tubulin [29] confirm its membrane-binding ability with a preference for DOPE lipid.

The reported orientation of the bound tubulin dimer on the membrane assures the accessibility of both α- and β-tubulin CTTs to block the VDAC pore embedded into the membrane [29,35]. CTTs-lacking tubulin-S do not produce characteristic blockages [34]. The similarity between the effects of tubulin-S on grA lifetime and conductance and the effects of the full-length tubulin found in the present work (Figure 4) confirm the previous results for tubulin-membrane binding [29], showing that tubulin CTTs are not involved in this interaction. This result also agrees with the previous observations of another group [70] and is consistent with the CTTs’ high negative charge. They represent only ~3% of the protein mass but carry ~40% of the total charge [71,72]. Considering that VDAC gating is known to be sensitive to bilayer properties [39,73,74], our data suggest that in addition to direct blocking of the VDAC pore by its CTT, tubulin could influence VDAC indirectly by modulating the mechanics of the surrounding channel bilayer.

A correlation between the thickness of the monounsaturated PC bilayers and grA lifetime has been reported previously [75] (reviewed in [47]). GrA lifetime decreased ~50 times when bilayer thickness was increased by 0.76 nm in DOPC compared with diC(22:1) (Table 1); this most likely arises due to the increase of the hydrophobic mismatch between the PC bilayer and grA dimer. DiC(22:1)PC membranes have a relatively thick hydrophobic region and thus require more deformation to accommodate a conductive grA dimer. For this reason, the channel lifetime is even shorter than that in pure DOPE (see Supplemental Figure S2 and Table 1). However, being a “PC lipid”, diC(22:1)PC forms membranes that are characterized by a rather dense headgroup packing, which does not allow for measurable tubulin binding (illustration in Figure 9). As a result, the addition of tubulin to the bulk solution does not affect integral mechanical properties of the bilayer appreciably and, consequently, does not change the channel lifetime. Very interestingly, tubulin does bind in the vicinity of the channel quite measurably, as is manifested by the channel conductance flickering and decrease in conductance (see Figure 5), thus definitely altering local membrane properties in close proximity to the gramicidin entrance; however, these properties turn out to be not as important as integral (global) ones.

The difference of ~0.4 nm in thickness between DOPC and DOPE bilayers shown in Table 1 (please note that data are available only for DOPE/DOPC (3:1) mixtures due to the inability of pure non-lamellar DOPE to form liposomes) can at least partially explain the 10-fold difference in grA lifetimes in these bilayers. This difference could be also be influenced by the previously suggested domination of grA peptide–lipid headgroup interactions over the effect of hydrophobic mismatch in regulating grA lifetimes [51].

GrA lifetime was also changed when monounsaturated acyl chains in PC lipid were replaced with phytanoyl chains in DPhPC. This branched chain lipid from archaebacteria (but not from mammalian membranes) has been routinely used by different labs in experiments with a variety of channels reconstituted into planar membranes [76,77,78,79,80] for the reason we have stated above: the high stability of the robust DPhPC planar bilayers that allow for long-lasting experiments and the application of high potentials. The GrA lifetime in DPhPC membranes was about two times longer than in DOPC membranes of similar bilayer thickness and identical polar headgroup (Table 1), but most striking was the effect of tubulin on the grA lifetime. We found that 30 nM of tubulin caused an approximately five-fold increase in channel lifetime in DPhPC, similar to its effect in pure DOPE membranes. These results could be interpreted by assuming that the branched phytanoyl chains of DPhPC entirely change PC membrane properties, making it DOPE-like, so that tubulin binds to DPhPC membranes with an efficiency similar to that of DOPE membranes. The grA lifetime in DPhPC is 20 times longer than in DOPE membranes (Table 1), suggesting that the mechanical properties of bilayers formed by branched DPhPC are different from those of DOPE membranes. However, the tubulin-binding propensity for DPhPC is close to that for DOPE because of the increased headgroup spacing in DPhPC. This conclusion is supported by the observations of Tristam-Nagle et al. [53], who, based on X-ray and neutron scattering and water permeability measurements on unilamellar vesicles formed from DPhPC and different phosphatidylcholines, suggested that from the biophysical perspective, DPhPC belongs to a different family of lipids than phosphatidylcholines with linear chain hydrocarbon chains. These authors showed that while the DPhPC membrane thickness of 3.64 nm is similar to that of the linear chain lipid DOPC membrane, the bending modulus of DPhPC is 30% smaller than that of DOPC. Regardless of the mechanism of the effect of DPhPC on grA channel parameters, tubulin’s effects on grA lifetime, conductance, and fast conductance fluctuations are due to the pronounced tubulin binding to the DPhPC membranes.

Taken together, our data suggest that tubulin-membrane binding is not defined by specific interactions of tubulin with lipid headgroups but rather depends on its lipid-dependent ability to induce unspecific distortions in the headgroup packing at the membrane surface.

### On the Nature of Tubulin-Induced grA Conductance Flickering

A distinctive feature of tubulin action on the grA channel is the generation of fast flickering with a characteristic time in a range of hundreds of microseconds in the channel current (Figure 1A, Figure 3, and Figure 5). Similar transient fast blocks of grA conductance have been reported already by a few groups [55,56,57,58] but with entirely different explanations of such phenomenon. It was first found that iminium ions (guanidium, acetalamidinium, formamide, and urea) at > 0.1 M concentration and in acidic pH induced well time-resolved grA conductance blockage events to the zero-current level with a characteristic blockage time in the range of 0.3–6 ms [55]. Importantly, grA lifetime was not affected by iminium ions, and block frequency was increased by a membrane potential increase. The phenomenon was explained as a direct blockage of K^+^ or H^+^ currents by iminium ions entering the channel [55]. A similar phenomenological behavior with a characteristic blockage time of ~0.1 ms was described for H^+^ conductance in native grA and in its covalently linked conducting dimers, with the latter demonstrating that fast current flickering is not related to the dissociation-association of grA monomers [56,57]. The intensity of current flickering correlated well with membrane thickness, which was varied by using different organic solvents for planar membrane formation from glyceryl monooleate using the “painted” membrane method. The intensity of the current blockage events increased with the membrane thickness [56]. The authors proposed that current fluctuations could be caused by bilayer undulations that produce transient obstructions to ion flow in the channel entrance, which is partially formed by the surrounding bilayer due to the significant hydrophobic mismatch between the grA dimer and bilayer. The deeper the grA dimer is embedded into the lipid bilayer, the more current blockage events occur [58].

Although K^+^ was the permeating cation in our experiments, the tubulin-induced grA closure events look very similar to those described above: the channel current was blocked to virtually zero (Figure 5A), the blockage frequency increased with applied potential (Figure 3C), and the characteristic blockage time was ~0.1–0.5 ms. To obtain some insight into the origin of these events, we performed experiments with membranes formed from diC(22:1)PC, in which the hydrophobic thickness was 0.76 nm larger than that of DOPC, 4.43 and 3.67 nm, respectively [61] (Table 1), to see if the frequency of channel blockages correlated with bilayer thickness [56]. We found that fluctuations between grA open and zero-conductance levels were well time-resolved in the current traces obtained in diC(22:1)PC membranes (Figure 5A). The power spectral densities in Figure 5B show a pronounced asymmetry in current blockages with respect to the sign of the applied voltage, which reflects a one-side tubulin addition (30 nM tubulin in the *cis* side) in our experiments. The density of flickering was highest when the positive potential was applied to the side of tubulin addition. Such asymmetry was also found in DPhPC and DOPC/DOPE membranes (Supplemental Figure S3). The highest spectral density, which corresponds to the highest frequency of blockage events, was obtained in the thickest diC(22:1)PC bilayers. These data can be understood in the framework of the interpretation proposed by Armstrong and Cukierman [56] if we are to assume that current blockages result from tubulin-enhanced bilayer undulations of the lipid funnel that forms the channel entrance. Alternatively, the tubulin dimer diffusing along the membrane surface could transiently block the channel conductance while approaching the lipid funnel. Within this model, the absence of the flickering of grA conductance in the presence of the α-tubulin membrane binding peptide is not surprising because of its drastically smaller size compared to a 100 kDa tubulin globule. The phenomenon of protein-induced fluctuations of grA conductance has been described previously using a system of gA channels modified by biotin on the C-terminus [59]. The addition of streptavidin induced fast channel flickering. The proposed mechanism of such flickering was ascribed to transient formations of transmembrane grA dimers and is specific to biotinylated grA channels crosslinked with streptavidin.

We obtained the kinetic characteristics of current blockages in diC(22:1)PC membranes using spectral analysis in combination with conductance measurements, as described previously. We found that the characteristic blockage time (*τ_off_*) calculated from the corner frequencies of Lorentzian spectra essentially does not depend on tubulin concentration (Supplemental Figure S4B) and is ~0.4 ms. However, the on-time (*τ_on_*) (the time between consecutive closure events) decreases with tubulin concentration (Supplemental Figure S4A). GrA conductance in diC(22:1)PC bilayers, averaged over different experiments, was insignificantly less than in DOPC, 19.1 ± 3.8 pS and 21.8 ± 0.4 pS, respectively (Table 1 and Supplemental Figure S2). The addition of 30 nM tubulin caused a ~15% conductance decrease, as measured in the same experiment (Figure 5 and Table 1). The decrease in the average conductance in the presence of tubulin could be accounted for by the high-frequency conductance blockages.

## 4. Material and Methods

Dioleoyl-phosphatidylcholine (DOPC), dioleoyl-phosphatidylethanolamine (DOPE), diphytanoylphosphatidylcholine (DPhPC), and dierucoylphosphatidylcholine (di(C22:1)PC) were purchased from Avanti Polar Lipids, Inc. Alabaster, AL. Gramicidin A (grA) was generously gifted from O. S. Andersen, Cornell University Medical College. Bovine brain tubulin was obtained from Cytoskeleton (Denver, CO, USA). The peptide corresponding to the α-tubulin H10 domain sequence (residues 328–346), VNAAIATIKTKRSIQFVDW, was synthesized in an FDA/CBER peptide synthesis core (White Oak, MD, USA) and dissolved in DMSO. All other chemicals were analytical grade.

### 4.1. Gramicidin A Measurements

Bilayer membranes were formed from monolayers as previously described [39]. Membrane-bathing solutions contained 1 M KCl buffered with 5 mM HEPES at pH 7.4. GrA was added from 1 nM ethanol solutions to both compartments of the cell [39]. After grA channels were reconstituted and their parameters were measured, tubulin was added to the *cis* side of the membrane under constant stirring for ~2 min. The potential is defined as positive when it is greater at the side of tubulin addition, defined as the *cis* side. Single-channel measurements were performed using an Axopatch 200B amplifier (Axon Instruments, Inc., Foster City, CA, USA) in the voltage clamp mode. Channel lifetime did not depend appreciably on the applied potential up to 200 mV for both DOPC and DOPE membranes. For the channel lifetime and conductance analysis, the signal was filtered by a low-pass Bessel filter at 10 kHz and saved into the computer memory with a sampling frequency of 50 kHz. Then, the records were digitally filtered at 0.1 kHz using the Bessel algorithm and analyzed using Clampfit 10.2 software as described previously [39] (Supplemental Methods). A digital Bessel filter at 2 kHz was used for the current noise power spectrum analysis. GrA channel lifetimes and mean conductances were collected and calculated as described previously [39] and in Supplemental Methods (see also Supplement Figure S6).

### 4.2. Bilayer Overtone Analysis (BOA) Measurements

BOA measurements were performed as described [29] (Supplemental Methods) using a Stanford Research Systems 830 lock-in amplifier and PLM made from DOPE:DOPC (4:1) (mol/mol) in 150 mM KCl buffered with 5 mM HEPES at pH 7.4, using the same protocol as the grA experiments. The intrinsic membrane potential, Ψ, measures the asymmetry between two lipid monolayer leaflets. Experimentally, Ψ was determined from the second harmonic of the bilayer current response to the applied voltage sinewave [63]. Data are presented as ΔΨ = Ψ − Ψ_t=0_, where positive ΔΨ corresponds to the increase of the positive charge on the *cis* surface of the membrane. Membrane capacitance and ΔΨ were measured once per minute using in-house Python 3.11 software [29]. Each data point is an average of 10 subsequent ΔΨ measurements taken when the signal reached a steady-state level, as defined by ΔΨ variations within ± 0.5 mV.

## 5. Conclusions

It was previously demonstrated that tubulin association with the membrane involves an α-helical amphiphilic domain of the α-tubulin subunit. In the bound state, the amphiphilic domain resides in the lipid headgroup region with a high preference for nonlamellar PE lipid. Here, using the grA channel as a sensitive molecular probe of bilayer mechanics, we showed that not only dimeric tubulin but also a synthetic peptide whose sequence corresponds to the membrane-binding helix H10 of α-tubulin binds to PE-containing planar membranes. They affect bilayer properties, which could be observed as an increase in the grA channel lifetime. These results indicate that both tubulin and the synthetic peptide reduce the bilayer deformation energy contribution at grA channel formation. Comparing lipids with different hydrophobic thicknesses and polar group compositions, we found that tubulin binding is sensitive to all bilayer parameters and most likely depends on the lipid packing stress distribution. Considering that the VDAC gating is known to be sensitive to lipid bilayer properties [39,73,74], our data suggest that in addition to direct blocking of the VDAC pore by tubulin CTTs, tubulin could influence VDAC properties indirectly by modulating the packing stress of the lipid bilayer. This is an example of a complex protein–membrane interaction where protein binding is regulated by lipids and, in turn, alters membrane properties and thus the functioning of other membrane proteins. Moreover, it is natural to expect that the effect of tubulin binding on membrane mechanics is reciprocal. The mitochondrial lipid composition is especially dynamic under apoptosis [81] or oxidative stress [82,83,84]. This allows us to suggest that mitochondrial membrane remodeling in many cellular processes—such as cell proliferation in disease and healthy development—modulates tubulin binding and, therefore, its regulatory interaction with VDAC.

## Data Availability

The data supporting the reported results can be obtained from Rostovtseva upon reasonable request.

## Supplementary Materials

The supporting information can be downloaded at https://www.mdpi.com/article/10.3390/ijms25042204/s1, References [29,39,63,85,86,87] are cited in the supplementary materials.
