# Supplementary material for: Dimeric Tubulin Modifies Mechanical Properties of Lipid Bilayer, as Probed Using Gramicidin A Channel
Source: Int J Mol Sci. 2024 Feb 12;25(4):2204. doi: 10.3390/ijms25042204 (PMC10889239; doi:10.3390/ijms25042204)
Supplement: Supplementary file 1 [file ijms-25-02204-s001.zip › ijms-2788601-supplementary.pdf]

SUPPLEMENTAL MATERIALS

Dimeric Tubulin Modifies Mechanical Properties of Lipid Bilayer as Probed by Gramicidin A Channel

Tatiana K. Rostovtseva[1], Michael Weinrich[2], Daniel Jacobs[1], William M. Rosencrans [1,3], and Sergey M. Bezrukov[1]

[1] Program in Physical Biology, *Eunice Kennedy Shriver* National Institute of Child Health and Human Development, NIH, Bethesda, MD, USA; rostovtt@mail.nih.gov
[2] NIST Center for Neutron Research, National Institute of Standards and Technology, Gaithersburg, MD, USA;
[3] Department of Biology and Biological Engineering, California Institute of Technology, Pasadena, CA, USA; wrosencr@caltech.edu

SUPPLEMENTAL METHODS

Gramicidin channels measurements and analysis

Bilayer membranes were formed from monolayers using membrane-forming lipid solutions of 5 mg/ml in pentane, across 70–90-μm diameter orifices in a 15-μm-thick Teflon partition that separated two compartments of ~ 1.2-1.5 ml volume [1]. The membrane potential was maintained using Ag/AgCl electrodes with 3 M KCl and 15% (w/v) agarose bridges.

GrA channel lifetimes were collected and analyzed as described previously [1] by fitting logarithmic single exponentials to logarithmically binned histograms [2] of at least 250 single-channel events. All lifetime histograms used 10 bins per decade. Fits to histograms used the Maximal Likelihood Estimator with the Simplex Algorithm in pClamp10.2. Conductance histograms used a bin width of 0.1 pA and were fitted using Gaussian distribution (Supplemental Figure S6). Each point in Figures 2, 4, and 7B is the mean value obtained in 3-5 experiments ± S.E. All measurements were made at room temperature T = 21 ± 1.0 °C.

BOA measurements and data analysis

The BOA measurements were performed as described [3,4] using a Stanford Research Systems 830 lock-in amplifier and planar lipid membranes made from DOPE:DOPC (4:1) (mol/mol) in 150 mM KCl buffered with 5 mM HEPES at pH 7.4. An excitation potential *V(t)* with frequency f_0 =

1933 Hz and amplitude $V_{ac} = 106\ mV$ ($75\ mV_{rms}$), and variable dc potential V_{dc} were applied to the membrane. The ac current was measured at the frequency of the second harmonic $2f_0 = 3866\ Hz$. For a lipid bilayer membrane with a capacitance C that scales with applied voltage as $C = C_0 + \alpha V^2$, where C_0 is the capacitance in the absence of an applied potential and α is related to the compressibility of lipid bilayer, the second harmonic current is [3,5,6]:

$$i_2(t) = -6\pi f_0 \alpha (\Psi + V_{dc}) V_{ac}^2 \sin(4\pi f_0 t). \quad (1)$$

Here Ψ, the intrinsic membrane potential, reports on the asymmetry between two monolayer leaflets. Experimentally, Ψ was determined from measuring $i_2(t)$ amplitude as a function of V_{dc}, which was swept from –50 to 50 mV in steps of 10 mV; the amplitude is minimized at $V_{dc} = -\Psi$.

SUPPLEMENTAL FIGURES

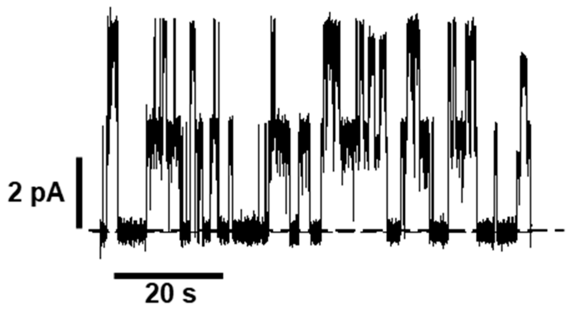

Supplemental Figure S1. Tubulin-S induces fast flickering of grA channel conductance characteristic for intact tubulin. Representative current traces of grA channels in DOPE membranes in the presence of 40 nM tubulin-S in the *cis* compartment. The applied voltage was 100 mV. The dashed line indicates zero current level. Other experimental conditions were as in Fig. 1.

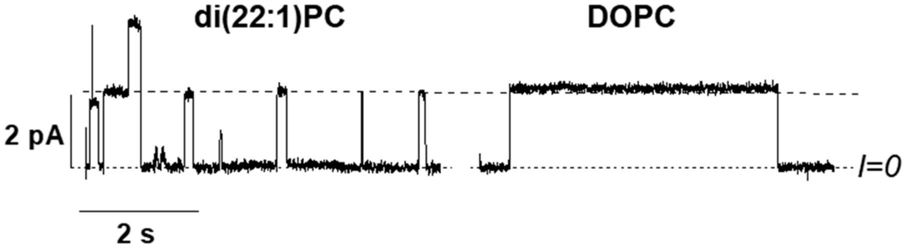

Supplemental Figure S2. The lifetime of grA channels is much shorter in di(22:1)PC bilayers than in DOPC. The representative current traces of grA channels were obtained in planar membranes formed from di(22:1)PC (*left trace*) or DOPC (*right trace*) in 1 M KCl, buffered with 5 mM HEPES at pH 7.4. The applied voltage was 100 mV. The dotted lines indicate zero current level; the dashed line indicates the current amplitude of grA channel in di(22:1)PC membrane. The grA channel conductance in DOPC is slightly higher (21.8 ±0.4 pS) than in di(22:1)PC (19.1 ± 3.8 pS).

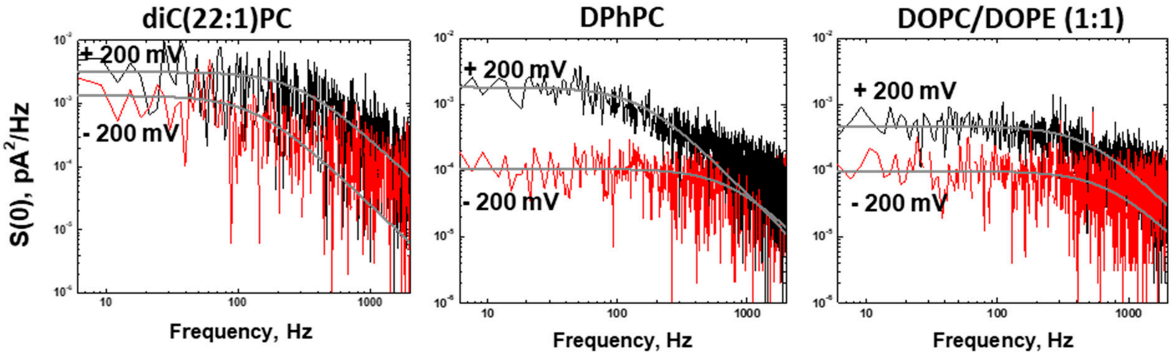

Supplemental Figure S3. Tubulin-induced current fluctuations in grA channel depend on the lipid composition and polarity of the applied voltage. Power spectral densities were obtained for single grA channels in di(C:22)PC, DPhPC, and DOPC/DOPE (1:1) membranes in the presence of 30 nM tubulin in the *cis* compartment. Solid lines represent the fits to Lorentzian spectra. The applied voltage was ± 200 mV. Current records were filtered with a digital 8-pole Bessel filter at 2 kHz.

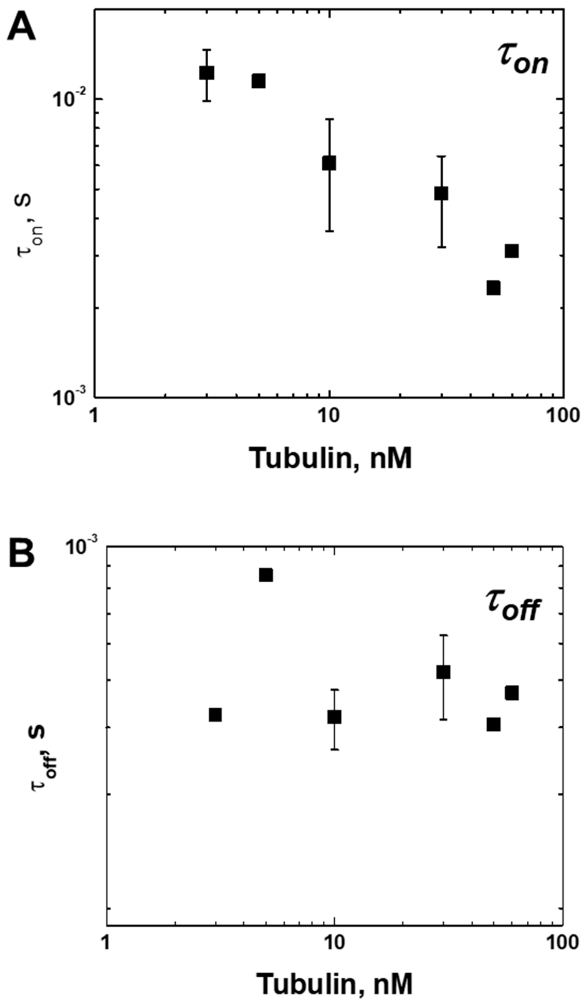

Supplemental Figure S4. Characteristic times of tubulin-induced blockage events in the grA channel conductance. The on-time – the time when channel stays open between blockage events - (τ_{on}) (**A**), and blockage time – the time when channel is closed - (τ_{off}) (**B**) were calculated from spectral analysis and conductance measurements. Membranes were formed from di(C:22)PC in 1 M KCl at pH 7.4 as in Fig. 5. 30 nM tubulin was added to the *cis* compartment. The applied voltage was +200 mV.

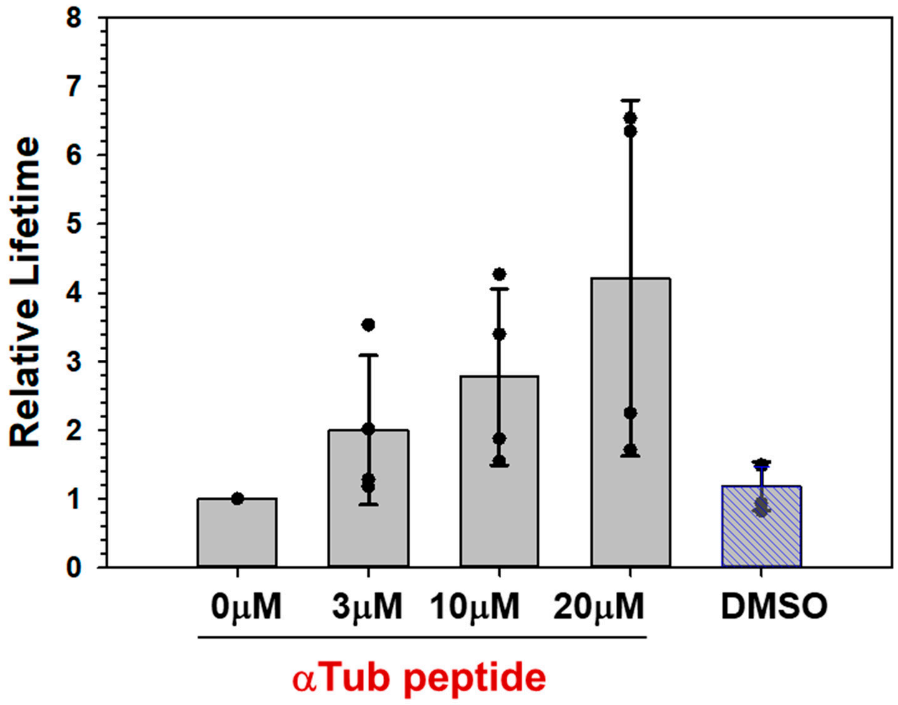

Supplemental Figure S5. GrA lifetime increases with α-tubulin peptide concentration. The relative grA lifetime at the given peptide concentration was calculated versus the lifetime without peptide for each individual membrane. The bars and error bars are the mean relative lifetimes and standard deviation from mean; the symbols represent data points of 4 independent experiments. Control measurements with addition of DMSO aliquots corresponding to 20 μM of α-tubulin peptide addition, show no effect on grA lifetime.

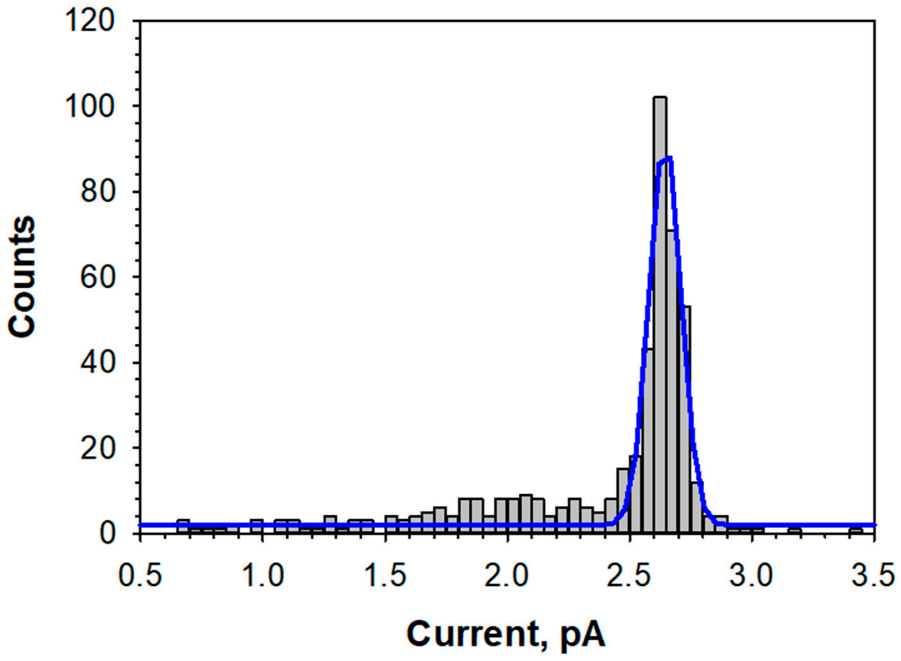

Supplemental Figure S6. Amplitude distribution of grA channel currents obtained in DOPE membrane in 1 M KCl at +100 mV applied voltage. The solid line is a fit to a Gaussian distribution with a main peak of 2.65 ± 0.06 pA. The pool of small or "mini" channels with conductances less than the 2σ of the main peak (typically < 25% of all events) is a characteristic of grA channels and is observed in any experimental condition.

Supplemental References

1. Rostovtseva, T.K., Kazemi, N., Weinrich, M., Bezrukov, S.M. Voltage gating of VDAC is regulated by nonlamellar lipids of mitochondrial membranes. *J Biol Chem*. **2006**, *281*, 37496-506.
2. Sigworth, F.J., Sine, S.M. Data transformations for improved display and fitting of single-channel dwell time histograms. *Biophys J*. **1987**, *52*, 1047-54.
3. Hoogerheide, D.P., Noskov, S.Y., Jacobs, D., Bergdoll, L., Silin, V., Worcester, D.L., Abramson, J., Nanda, H., Rostovtseva, T.K., Bezrukov, S.M. Structural features and lipid binding domain of tubulin on biomimetic mitochondrial membranes. *Proc Natl Acad Sci U S A*. **2017**, *114*, E3622-E3631.
4. Peterson, U., Mannock, D.A., Lewis, R.N., Pohl, P., McElhaney, R.N., Pohl, E.E. Origin of membrane dipole potential: contribution of the phospholipid fatty acid chains. *Chem Phys Lipids*. **2002**, *117*, 19-27.
5. Carius, W. Voltage Dependence of Bilayer Membrane Capacitance - Harmonic Response to Ac Excitation with Dc Bias. *Journal of Colloid and Interface Science*. **1976**, *57*, 301-307.
6. Sokolov, V.S., Kuz'min, V.G. [Measurement of differences in the surface potentials of bilayer membranes according to the second harmonic of a capacitance current]. *Biofizika*. **1980**, *25*, 170-2.
